# A Comprehensive Review of Emerging Therapies for Type 2 Diabetes and Their Cardiovascular Effects

**DOI:** 10.7759/cureus.65707

**Published:** 2024-07-29

**Authors:** Arnaldo J Acosta G, Eesha Chitneni, Claudia Jeanette Manzanares Vidals, Sravani Modumudi, Sobia Hammad, Ashee Verma, Rahul Y Rajesh, Aimen Khaliq, Olaoluwa Adeyemi, Farhat Majeed, Rucha V Gujar

**Affiliations:** 1 Internal Medicine, Hospital Universitario Dr. Alfredo Van Grieken, Coro, VEN; 2 Internal Medicine, MediCiti Institute of Medical Sciences, Hyderabad, IND; 3 Internal Medicine, General Hospital of Toluca Dr. Nicolas San Juan, Toluca, MEX; 4 Internal Medicine, Kamineni Academy of Medical Sciences and Research Center, Hyderabad, IND; 5 Medicine, Jinnah Medical and Dental College, Karachi, PAK; 6 Internal Medicine, Ruxmaniben Deepchand Gardi Medical College, Kota, IND; 7 Internal Medicine, Tbilisi State Medical University, Tbilisi, GEO; 8 Medicine, Liaquat National Hospital and Medical College, Karachi, PAK; 9 Internal Medicine, Richmond Gabriel University, Kingstown, VCT; 10 General Medicine, Quaid-e-Azam Medical College, Bahawalpur, PAK; 11 Internal Medicine, Sir H. N. Reliance Foundation Hospital and Research Centre, Mumbai, IND

**Keywords:** ischemic heart diseases, advanced therapy, cardio vascular disease, diabetes mellitus, glp-1-r agonists, sglt2 inhibitors

## Abstract

The discovery of inhibitors for sodium-glucose cotransporter 2 (SGLT2) and glucagon-like peptide-1 receptor agonists (GLP-1 RA) has significantly improved type 2 diabetes management. Large-scale clinical studies have shown that both SGLT2 inhibitors and GLP-1 RA enhance cardiovascular health. Benefits include reduced cardiovascular disease risk, lower mortality, fewer heart failure hospitalizations (SGLT2 inhibitors), and stroke prevention (GLP-1 RA). Additionally, these drugs slow chronic kidney disease progression. This comprehensive treatment targets vascular events. Despite differences, both drug classes are crucial. GLP-1 RA mainly reduce stroke risk, while SGLT2 inhibitors alleviate heart failure. Our findings, based on a literature review, will address the renal and cardiac effects of SGLT2 inhibitors and GLP-1 RA in both diabetics and non-diabetics, highlighting their combined benefits for heart conditions.

## Introduction and background

Cardiovascular complications, including myocardial infarction, stroke, heart failure, and death, are more common in those with type 2 diabetes. Diabetes increases the risk of cardiovascular disease and other causes of death for one-third of men and 50% of women [1]. From an anticipated 537 million in 2021, the number of individuals living with diabetes is anticipated to rise to 643 million by 2030 [2]. Treatment for type 2 diabetes is further complicated by cardiovascular disease. It is critical for people with type 2 diabetes to have a better understanding of the many ways the condition may be managed in order to reduce their future risk of cardiovascular disease. Recently developed methods for managing diabetes mellitus have far-reaching consequences for the cardiovascular system. Thus, they are not without their advantages and disadvantages. According to many large-scale studies examining cardiovascular outcomes, new glucose-lowering medicines, such as sodium-glucose cotransporter 2 (SGLT2) inhibitors and glucagon-like peptide-1 receptor agonists (GLP-1 RA), significantly reduce the likelihood of serious adverse cardiovascular events and other outcomes related to cardiovascular health, such as acute cardiac arrest. New approaches to managing type 2 diabetes have emerged in response to the results of these studies. Those at high risk for atherosclerotic cardiovascular events due to type 2 diabetes are presently recommended by endocrinology and cardiology guidelines to take GLP-1 RA [3]. This recommendation demonstrates progress towards a more comprehensive approach that prioritizes the prevention of cardiovascular problems in type 2 diabetics, as opposed to a narrow emphasis on glucose metabolism. Simultaneously, in follow-up studies evaluating cardiovascular outcomes in type 2 diabetic mellitus patients predisposed to cardiovascular complications, alogliptin, saxagliptin, and sitagliptin were performed similarly to placebo. A higher-than-expected risk of hospitalization for heart failure was associated with saxagliptin treatment. Thiazolidinediones, including rosiglitazone, increase the risk of ischemic cardiovascular events, according to research that used meta-analyses of randomized controlled trials [4]. Examining the various effects of contemporary anti-diabetic medications on the cardiovascular system is the goal of this review. This study takes a close look at the statistical data about these medications and how they affect the diabetes patient group. Taking advantage of every opportunity to decrease the risk of cardiovascular events is critical for people with type 2 diabetes mellitus since their risk is already high. There has been progress in reducing diabetes-related deaths and improving the management of cardiovascular risk factors, but overall, the number of deaths in people with diabetes is still higher than in the general population, and CVD is still the leading cause of death and disability in the diabetic population [5]. The insatiable curiosity about diseases and their impact on various bodily systems, as well as the management of their symptoms, has driven scientific inquiry and the development of treatments that are not only effective against the sickness at hand but also mitigate its long-term consequences. An estimated 38.4 million individuals worldwide, across all age groups, are living with diabetes, and this number is rising rapidly in every nation. With both known and undiagnosed cases, 11.6% of the United States population will be living with diabetes in 2021 [6].

Diabetic vascular scarring, most noticeably in the kidneys, leads the body to retain water and salt, which in turn raises blood pressure, the devastating consequence of diabetes on the cardiovascular system. Diabetes and its devastating effects on the cardiovascular system have both been greatly ameliorated by modern therapeutic advances, such as the ability to instantly regulate blood glucose levels (with inhaled insulin) and to do so for an extended period of time (with weekly injections) [7]. Additional evidence for the indication was provided in the 2021 ESC Guidelines for the Prevention of Cardiovascular Disease, which recommended GLP-1 RA for individuals with type 2 diabetes and atherosclerotic cardiovascular disease to lower the risk of cardiovascular disease and renal consequences. Although they are still in their infancy as a treatment option for type 2 diabetes, SGLT2 inhibitors have quickly become one of the most popular options owing to their ability to reduce cardiovascular risks associated with the disease and their success in controlling blood glucose levels.

New diabetic treatments are being studied for their potential effects on the cardiovascular system, the endothelium, the smooth muscle of blood vessels, platelets, and immune system cells. This article covers a lot of ground in this area. Our attention was drawn to their role in the formation of atherosclerotic plaques. We are hoping for further clinical use of these medicines due to their effectiveness as cardiovascular prevention measures.

## Review

Diabetes and cardiovascular disease

There is a high rate of mortality and morbidity linked to cardiovascular disease and diabetes, two of the top causes of death worldwide [8]. The fact that these illnesses often co-occur in the same patient adds to the healthcare load and the individual risk of mortality and morbidity. Numerous cardiovascular illnesses, such as coronary artery bypass grafting, peripheral artery disease, and insulin resistance, all have a common ally in chronic hyperglycemia and other metabolic abnormalities associated with diabetes, such as dyslipidemia, hypertension, obesity, and insulin resistance [9,10].

People with diabetes alone were twice as likely to suffer from a variety of cardiovascular diseases as those with other prevalent risk factors [11]. The World Health Organization reports that 17.9 million people died in 2019, with cardiovascular disease accounting for 32% of all mortality globally. More than one-third of all fatalities in 2021 were attributable to cardiovascular disease, which killed 2.5 million individuals. A substantial rise occurred in 1990 when 12.1 million fatalities were attributed to cardiovascular disease. Conversely, high-income nations have seen a far quicker decline in the mortality toll from cardiovascular disorders. The World Heart Federation warns that global progress in avoiding cardiovascular disease is either about to come to a halt or has already happened, highlighting the need for concerted measures to reverse the trend [12,13]. Recent statistics from the International Diabetes Federation show that 537 million people (10.5% of the adult population) between the ages of 20 and 79 had diabetes in 2021, with almost half (44.7%) remaining undiagnosed. The International Diabetes Federation predicts that the global diabetes population will reach 783 million in 2045, up from 643 million in 2030 [14].

Nearly 90% of people with diabetes have type 2. The first-line glucose-lowering drug used to treat type 2 diabetes is metformin, and lifestyle adjustments are the mainstay of treatment. However, there are no cardiovascular advantages to metformin [15]. Cardiovascular disease affects over 32.2% of the global population with type 2 diabetes. Cardiovascular disease accounts for about half of all deaths in people with type 2 diabetes. Despite ongoing efforts, no nation or healthcare system has been able to eliminate the risk of type 2 diabetes mellitus. Despite a decline in cardiovascular disease incidence and mortality rates in high-income nations, these nations account for a mere 10% of the global population. The prevalence and causes of cardiovascular disease in people with type 2 diabetes in low- and middle-income nations are not well understood [16].

Though the incidence and mortality rate of cardiovascular diseases connected to type 2 diabetes have decreased, the prevalence and death rate of these illnesses in people with type 2 diabetes remain on the rise. However, most type 2 diabetes-related cardiovascular diseases are preventable with dietary and pharmaceutical adjustments. Its progressive character is taken into account by second-line agents. Although sulfonylureas are beneficial, they may lead to hypoglycemia and weight gain [17]. To avoid cardiovascular illness in type 2 diabetics, it is best to use relatively recent medications such as GLP-1 agonists and SGLT2 inhibitors. To improve the therapeutic approach for type 2 diabetic patients with preexisting cardiovascular disease, new drugs have been developed that not only achieve glycemic control but also induce weight loss. These drugs include GLP-1 agonists, SGLT2 inhibitors, blood pressure and lipid-lowering medications, and others.

**Figure 1:**
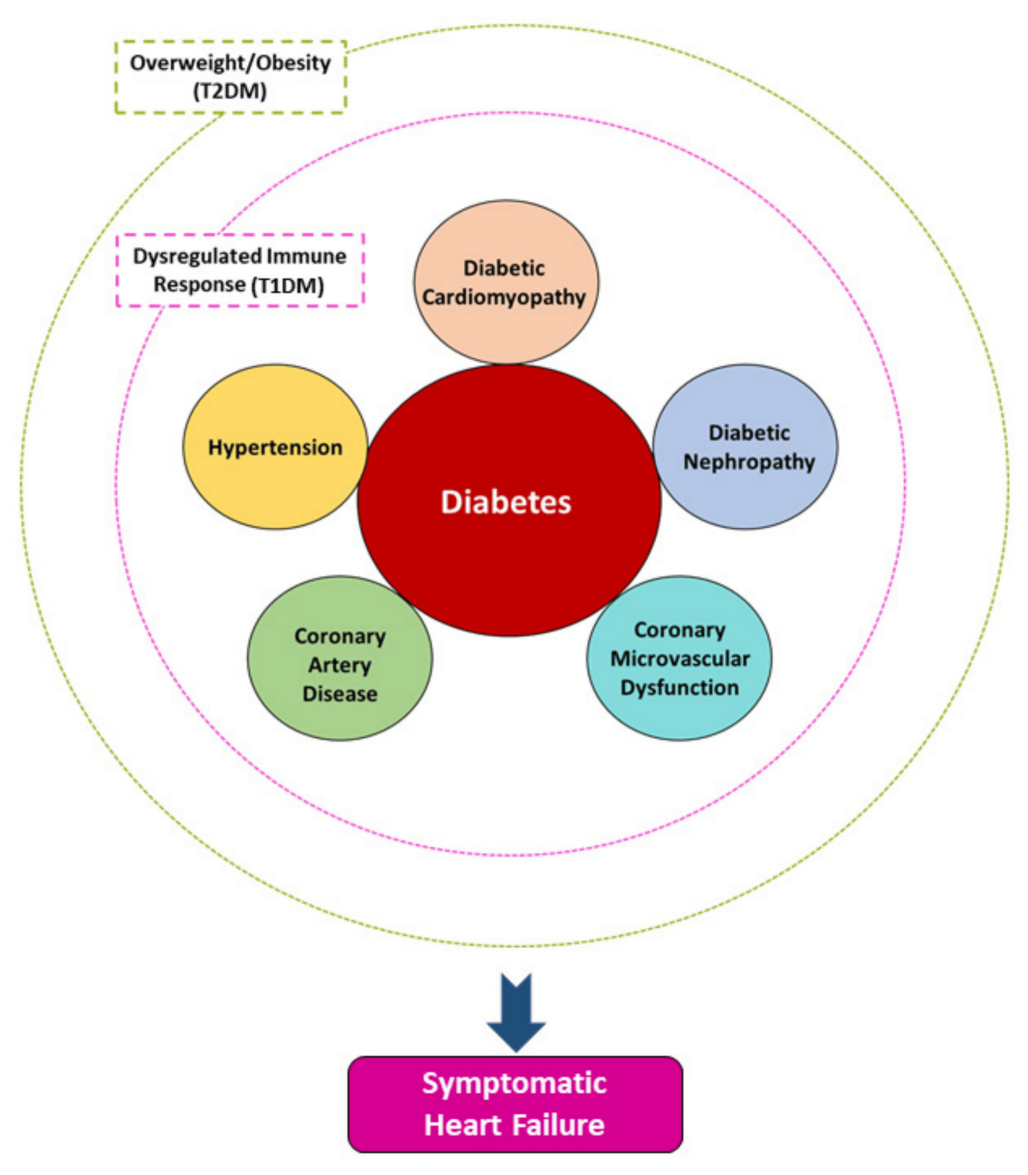
The development of heart failure in individuals with diabetes mellitus (DM) is a complex process that is primarily caused by the harmful impact of high blood sugar levels (hyperglycemia) and its metabolic effects on the heart muscle (known as diabetic cardiomyopathy). Other factors such as hypertension, coronary artery disease, coronary microvascular dysfunction, and diabetic nephropathy often coexist and contribute to the development of heart failure. In individuals diagnosed with type 1 DM, heart failure (HF) occurs due to an imbalanced immunological response. Meanwhile, in the majority of individuals with type 2 DM, heart failure develops in the presence of excess weight or obesity Reproduce from reference [21], under the terms and conditions of the Creative Commons Attribution (CC BY) license (https://creativecommons.org/licenses/by/4.0/). Copyright © 2021 by the authors. Licensee MDPI, Basel, Switzerland.

The development of novel medications that lower blood glucose levels has led to a shift in how type 2 diabetics are being treated. Results from cardiovascular endpoint trials indicate the cardioprotective benefits of SGLT2 inhibitors and GLP-1 RA that are independent of HbA1c [18,19]. A reduced risk of chronic renal disease and heart failure is associated with drugs that suppress SGLT2. While SGLT2 inhibitors primarily reduce pre- and after-load on the heart, newer data suggest that GLP-1 RA may also have anti-inflammatory and plaque-stabilizing effects [20]. As a result of these studies and findings, the clinical advice and guidelines for treating type 2 diabetes have changed. People who are at high or very high risk of cardio-renal complications should take medication to protect their hearts, such as SGLT2 inhibitors or GLP-1 RA, although this assessment may be done at baseline. Patients who are at high risk for chronic renal disease and chronic heart failure, or who have both diseases, should take SGLT2 inhibitors (Figure 1) [21].

Inhibitors of the SGLT2 cotransporter

**Figure 2:**
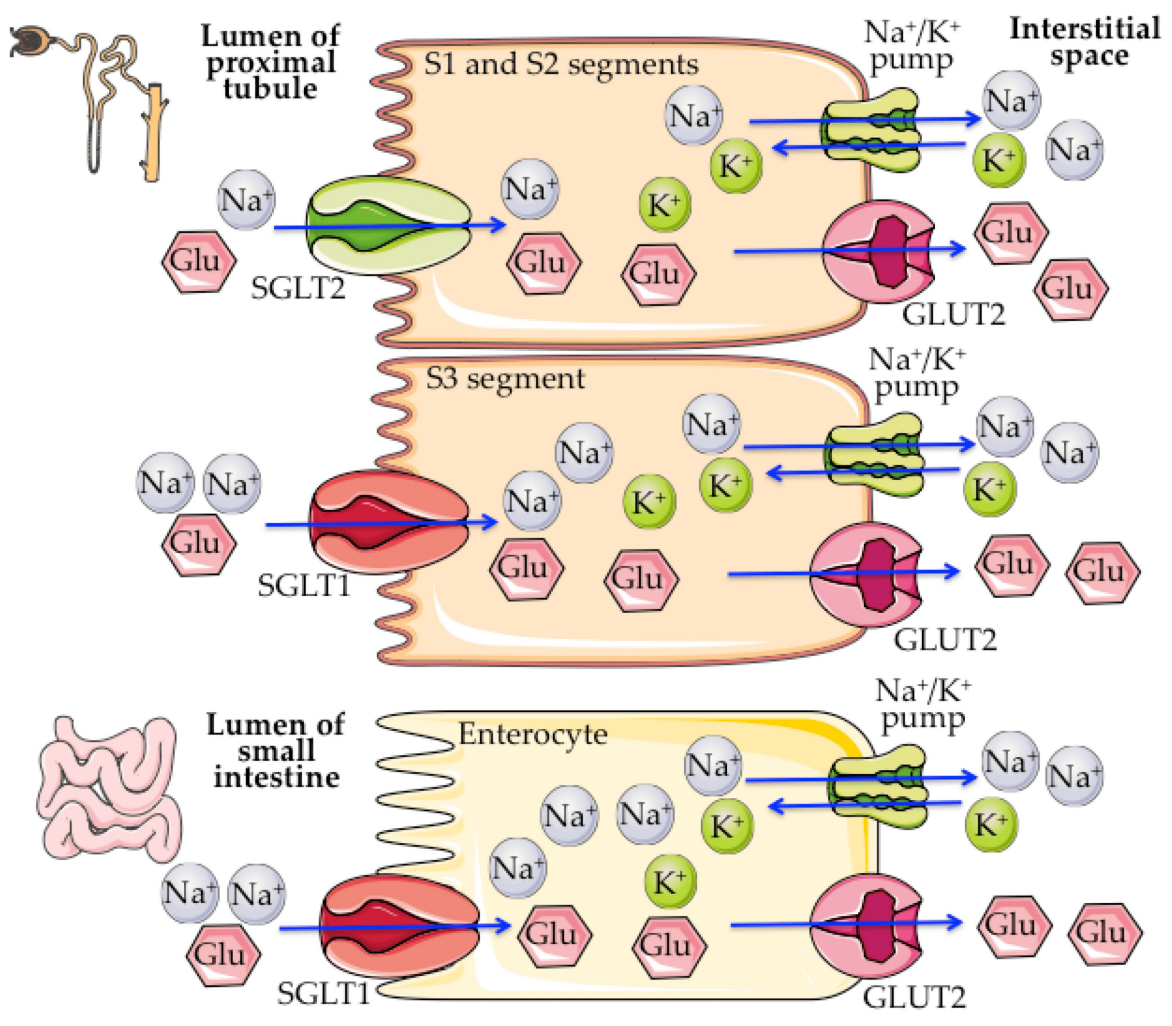
The primary locations where SGLT1 and SGLT2 are expressed and their respective functions SGLT1: Sodium/glucose cotransporter-1, SGLT2: Sodium-glucose cotransporter-2, Glu: Glucose, GLUT2: Facilitated glucose transporter member 2, Na+: Sodium ions, K+: Potassium ions Reproduce from reference [24], under the terms and conditions of the Creative Commons Attribution (CC BY) license (https://creativecommons.org/licenses/by/4.0/). Copyright © 2022 by the authors. Licensee MDPI, Basel, Switzerland.

Originally isolated from apple tree root bark, the phenolic glycoside known as phlorizin was the first SGLT2 inhibitor [22]. Its antipyretic properties were formerly thought to have been identified and isolated in the 19th century. After further investigation, it was shown that, when given to dogs, phlorizin causes glucosuria, which is similar to diabetes. The symptoms of this illness include a decrease in appetite, an increase in urine output, and the presence of glucosuria. The proximal tubule's role in renal glucose reabsorption was first described in the 1960s. The SGLT2 cotransporter was successfully cloned in the 1990s. Because of this, we now know more about the pharmacological effects of phlorizin and the kidneys' glucose-handling mechanisms. Researchers saw the reduction of renal glucose reabsorption as a possible target for diabetes management and set out to examine it. While phlorizin had no effect on insulin action in healthy rats, it increased insulin sensitivity in diabetic rat models, according to preclinical studies done in the 1980s [23]. Figure 2 presents the primary locations where SGLT1 and SGLT2 are expressed and their respective functions [24].

Because phlorizin is not absorbed by the body when taken orally, the only effective method of delivery is intravenous. The first SGLT2 inhibitor that could be taken orally, T-1095, was developed in the 1990s. Results showed that the treatment reduced microalbuminuria, helped rats lose weight, and improved their HbA1c levels [25]. T-1095 had serious gastrointestinal side effects and intolerance due to its action on intestinal SGLT1, and it was not selective towards SGLT2. The development of at least seven different oral SGLT2 inhibitors began with T-1095. Dapagliflozin, empagliflozin, and canagliflozin are the three that have been approved by the FDA. When compared to SGLT1, all three of them strongly favor limiting SGLT2.

The benefits of SGLT2 inhibition in high-risk patients for cardiovascular difficulties have been shown in recent treatment trials with empagliflozin, canagliflozin, and dapagliflozin [26,27]. In both diabetic and non-diabetic renal illnesses, when administered in addition to standard therapy, they significantly reduce cardiovascular and all-cause mortality, heart failure hospitalizations, adverse cardiovascular events, and albuminuria development. One important finding is that SGLT2 has been effective in treating renal illness and heart failure independently of diabetes. In order to comprehend the rationale behind the wide variety of therapeutic benefits they give, it is vital to get a full knowledge of the direct and indirect physiological processes and consequences of SGLT2 inhibition [28,29].

Physiological impact of SGLT2 blockage

Improved Glucose Control

Glucose is excreted in the urine, known as glucosuria, when the SGLT2 cotransporter is blocked. By inhibiting SGLT2 cotransporter, gliflozin-containing medications stop glucose reabsorption in the proximal tubule's S1 and S2 segments. As a consequence, the renal threshold for glucosuria drops, and TmaxG drops to about 40-80 mg/dL. Because glucosuria causes a significant loss of energy, SLGT1 cotransporters react by increasing reabsorption to around 40% [30]. This was shown in a rat preclinical study, which found that, compared to mice with a single SGLT2 knockout, those with a double deletion of SGTL1 and SGLT2 produced much more glucosuria. A higher risk of hypoglycemia is not significantly associated with glucose level management with SGLT2 inhibitors. Lessons in glycemic control are shown by a 0.5-1% drop in HbA1c levels [31]. When considering the effects of SGLT2 inhibition on diabetes treatment, it is important to note that this leads to enhanced insulin responsiveness and beta-cell activity. Efficacy in glucose control has been shown in the main clinical trials using SGLT2 inhibitors on many occasions [32]. This benefit remains even when used in conjunction with more traditional forms of therapy.

**Figure 3:**
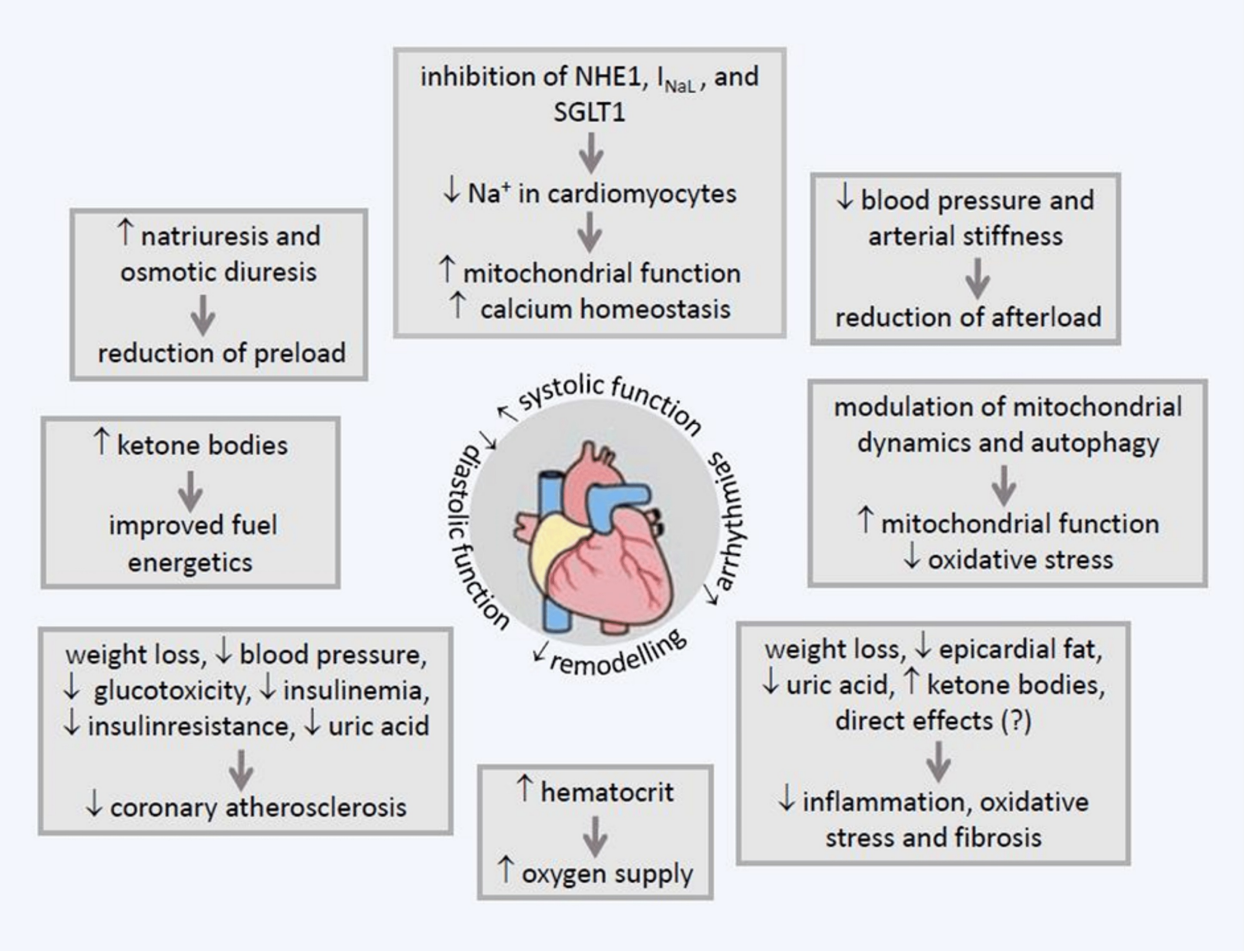
The main pleiotropic effects of SGLT2 inhibitors on the heart Reproduce from reference [33], under the terms and conditions of the Creative Commons Attribution (CC BY) license (https://creativecommons.org/licenses/by/4.0/). Copyright © 2022 by the authors. Licensee MDPI, Basel, Switzerland.

An increase in the excretion of salt in the urine is called natriuresis. Reversal of stimulation of tubulo glomerular feedback and improvement of blood pressure are two of its related effects. Natriuresis, marked by a decline in salt and water balance, is another complication of SGLT2 inhibition, together with glucosuria. As the plasma volume decreases, the systolic and diastolic blood pressures drop by 3-6 mmHg and 1-1.5 mmHg, respectively (Figure 3) [33].

Secondary Effects of SGLT2 Inhibition

Raising the albuminuria level: Results from trials including both diabetic and non-diabetic patients with CKD demonstrate that SGLT2 inhibitors significantly reduce albuminuria [29]. Both this effect and the effect of the renin-angiotensin-aldosterone system (RAAS) blockage are independent and cumulative. Several causes contribute to the reduction in albuminuria. One is the improvement in general blood pressure. Another is the constriction of the blood vessels leading to the kidney, which lowers the pressure within the filtering units of the kidney and increases the filtration rate. Evidence from many studies suggests that podocytes, which include SGLT2 cotransporters, may benefit from SGLT2 inhibition. By improving glucotoxicity and restoring insulin sensitivity, dapagliflozin and empagliflozin may reduce podocyte dysfunction and effacement [34]. After six to 12 months of medication, SGLT2 inhibitors result in a weight loss of 2-4 kg. Volume loss from constriction causes the first weight loss, followed by calorie loss via glucosuria. When management of diabetes begins with the goal of weight loss, the American Diabetes Association recommends SGLT2 inhibitors. A state of relative glucose deficit is caused by SGLT2 inhibition and the subsequent glucosuria, which in turn causes a shift in the utilization of energy sources towards lipids. Because of this, the detrimental consequences of cell lipid excess are reduced, and the body's resistance to oxidative stress is improved. An increase in ketone production, which provides a more desirable energy source for cardiac and renal cells, is another benefit of this [35].

Improved transport of oxygen and the deficit of hemoglobin: As indicated earlier, the improved performance of proximal tubular cells and the reduction in energy consumption cause both the demand for and supply of oxygen in the cortex to decrease [36]. The SGLT1 cotransporters located in the latter part of the proximal tubule reabsorb glucose that is given later, which causes the renal outer medulla to spend more energy and use more oxygen. An increase in erythropoietin synthesis is caused by hypoxia, which activates hypoxia-inducible factors HIF1 and HIF2. In addition, an increase in hemoglobin levels facilitates the delivery of oxygen to different tissues as a result of a small volume reduction and a small narrowing of blood vessels. Clinical trials have shown that patients treated with SGLT2 inhibitors have improved hemoglobin levels. It seems that dapagliflozin enhances erythropoiesis by inhibiting hepcidin and other proteins involved in iron metabolism [37,38].

Possible Extra Repercussions

The levels of inflammatory markers, such as toll-like receptor (TLR-4), interleukin-6 (IL-6), interferon- γ (IFNγ), transforming growth factor-beta (TGF-β), nuclear factor kappa β (NF-κβ), tumor necrosis factor (TNF), and transforming growth factor-beta (TGF-β), have been seen to be decreased by gliflozins [39,40]. The amount of myofibroblasts in heart tissue is reduced, mesangial development is reduced, and mitochondrial function is enhanced [41]. According to the study, empagliflozin seems to reduce the IL-β inflammatory pathway in cells located near the tubules. Taking these measures would reduce oxidative stress, inflammation, and fibrosis in renal and cardiac tissue [42]. These additional changes are essential, but the metabolic and hemodynamic consequences of SGLT2 inhibition seem to be more crucial.

Potential mediators of cardioprotection and GLP-1 RA in pre-clinical investigations of ischemic heart disease

In preclinical animals, GLP-1 RA often protect against myocardial ischemia/reperfusion injury, which is consistent with results seen with SGLT2 inhibitors. Intravenous administration of natural GLP-1 to male Sprague-Dawley rats (4.8 pmol/kg/min) produced the first beneficial effects. For 30 minutes, these rats had their left anterior descending (LAD) coronary artery temporarily blocked, and then they were reperfused for two hours. This therapy considerably reduced the infarct size. However, keep in mind that the brief reperfusion period could cause some worries. Exenatide and other GLP-1 RA have shown positive results in several trials. Exenatide was given to Dalland Landrace pigs at a dosage of 10 μg five minutes before reperfusion in one study and then again at a dose of 10 μg twice a day for three days. After three days of reperfusion and a 75-minute left circumflex coronary artery closure, this therapy reduced the infarct [43]. Albiglutide was administered orally to male Sprague-Dawley rats for three days in 2011 research by Müller et al. [43] at dosages of 1, 3, or 10 mg/kg. A 30-minute LAD coronary artery blockage and 24 hours of reperfusion followed this therapy in rats. A significant decrease in infarct size was shown by the data. A rise in myocardial glucose oxidation is linked to albiglutide-induced protection against ischemia/reperfusion injury. One possible method by which GLP-1 RA protect the heart is by increasing glucose oxidation. The infarct size and severity of ischemia/reperfusion damage may be directly mitigated by increasing myocardial glucose oxidation [44]. The GLP-1 RA liraglutide only increases glucose oxidation rates in functioning, isolated mouse hearts when the hearts are removed from mice that have previously received systemic liraglutide treatment, according to the research [45]. The isolated working heart does not show an increase in glucose oxidation rates when given liraglutide directly. The metabolic results provide evidence that administering liraglutide to male C57BL/6J mice at a dosage of 200 μg/kg improves their functional cardiac recovery following 30 minutes of global no-flow ischemia and 40 minutes of reperfusion in the Langendorff mode. On the other hand, researchers found no benefit when liraglutide (at a dose of 30 nM) was administered directly to the isolated heart.

Reduced cell death in the heart muscle may be one benefit of using GLP-1 and GLP-1 RA to lessen the severity of a heart attack and its aftermath. Evidence of reduced apoptosis has been found to be supported by a number of indicators of cell death, including cleaved caspase 3 levels and terminal deoxynucleotidyl transferase dUTP nick end labeling [46]. It seems that GLP-1 R is absent from mouse cardiomyocytes. The effects of GLP-1 RA on myocardial energy metabolism have been previously shown to be consistent with this. Thus, it is likely that GLP-1 RA's anti-apoptotic effects are mediated indirectly. An increase in insulin and a decrease in glucagon in the blood may explain why myocardial glucose oxidation is stimulated and cardiomyocyte apoptosis is reduced. Unfortunately, no research has shown a conclusive association between changes in hormone secretion and these effects; therefore, it is unclear whether this is the direct source of these effects [47]. An important factor in ischemia/reperfusion damage is caveolins, which are membrane proteins necessary for the production of caveolae (membrane invaginations). In addition, there is some indication that they may have a role in the reductions in infarct size that are caused by GLP-1 RA. A study found that exendin-4 significantly reduced infarct size and circulating cardiac troponin I levels in male C57BL/6 mice between the ages of eight and 10 weeks. The LAD coronary artery was blocked for 30 minutes, and then the mice were reperfused for two hours. Interestingly, male mice deficient in caveolin-3 did not engage in any of these behaviors. The findings were confirmed in male C57BL/6 mice that received 30 ng/kg of exendin-4 intravenously before the LAD coronary artery was blocked for 30 minutes and then reperfused for two hours. Caveolin-3 was shown to travel more freely to buoyant caveolar fractions as a result [48].

The cleavage product GLP-1 (9-36), which is regulated by DPP4, is another potential mechanism by which GLP-1 protects the heart. Several studies have shown that GLP-1 (9-36) has effects on the cardiovascular system, contradicting the initial belief that it has no biological impact. More specifically, research has shown that isolated Langendorff rat hearts and live canines exhibit an increase in glucose absorption after receiving a direct dose of GLP-1 (9-36) [49]. Furthermore, isolated mesenteric arteries that had been previously narrowed with phenylephrine may be widened by administering GLP-1 (9-36). Furthermore, GLP-1 (9-36) can be converted to GLP-1 (28-36) by neutral endopeptidase 24.11. This new form of GLP-1 may have an effect on mitochondria and oxidative stress reduction on an individual level. Reducing the effects of ischemia and reperfusion injuries is compatible with these measures. This cardioprotective mechanism is intriguing because it could explain why some studies have shown protective responses when cardiac myocytes or isolated hearts are treated directly, even though these cells do not express GLP-1R. Since DPP4 inhibitors block GLP-1 production (9-36) and, by extension, GLP-1 (28-36), these findings may also explain why they are ineffective in cardiovascular outcome trials (CVOTs). However, the fact that the majority of GLP-1 RA that have performed well in cardiovascular outcome trials are resistant to cleavage by dipeptidyl peptidase-4 inhibitor (DPP4) casts doubt on the relevance of this mechanism. In contrast to GLP-1, liraglutide may be hydrolyzed by neutral endopeptidase 24.11 and DPP4, leading to distinct cleavage products. Whether these cleavage products can have the same effects as GLP-1 (9-36) and GLP-1 (28-36) is not yet known [50,51]. Similar issues to those mentioned for SGLT2 inhibitors also apply to GLP-1 RA, despite the fact that there is a lot of congruence between clinical and preclinical studies. Cardiovascular outcome trials for GLP-1 RA now include people who often use βblockers and other cardiovascular medications. Preclinical studies may not be as applicable as intended due to these medications. Further, animals free of diabetes have been the primary subjects of the preclinical investigations.

Currently under development agents

Agonists for the Amylin/GLP-1 Dual Receptors

In the pancreas, islet cells secrete the hormone amylin. Beta cells secrete it in tandem with insulin when nutrients are consumed. It delays the emptying of the stomach by blocking the secretion of glucagon after eating. For both monotherapy and weekly dosing with the long-acting GLP-1 RA semaglutide, cagrilintide is a long-acting counterpart of amylin that is administered subcutaneously [52]. A 32-week trial, including 92 people with type 2 diabetes with a body mass index of 27 kg/m^2^ or above, examined the efficacy of once-weekly administration of carberisema, semaglutide, or cagrilintide. The trial was carried out at many centers. Everyone involved in the study was in the dark about which therapy each person was getting since it was a double-blind trial. As an intermediary step in determining whether or not the medications were safe and effective, this investigation was carried out during phase 2. On average, HbA1c levels changed by 2.2% for CagriSema, 1.8% for semaglutide, and 0.9% for cagrilintide between the beginning of the research and week 32. The average reduction in body weight from the beginning to week 32 with CagriSema, semaglutide, and cagrilintide is -15.6%, -5.1%, and -8.1%, respectively [53].

Triple-Receptor Agonists for GIP, Glucagon, and GLP-1

In the pancreatic islets, alpha cells secrete glucagon, a 29-amino-acid peptide. Encouraging glycogenolysis and gluconeogenesis is its principal role. Increasing blood glucose levels is the physiological role of glucagon. Treatment for type 2 diabetes should aim to reduce, rather than increase, the effects of glucagon. The effects of glucagon on hunger, satiety, and energy expenditure have been the subject of several studies. Retatrutide is a weekly intravenous injection that acts as a triple hormone agonist at the GIP/GLP-1/glucagon receptor [43]. People who have been diagnosed with type 2 diabetes were recruited as participants for the phase 2 project. The participants were put into one of six groups and given weekly injections of either a placebo, 1.5 mg dulaglutide, or retatrutide at various maintenance doses: 0.5 mg, 4 mg (with a starting dosage of 2 mg), 4 mg (without escalation), 8 mg (with a starting dose of 2 mg), 8 mg (with a starting dose of 4 mg), or 12 mg (with a starting dose of 2 mg). The orders were drawn at random. At 24 weeks, the following are the mean subtractions from baseline in HbA1c: -0.01%, -1.41%, -0.43%, -1.39%, -1.30%, -1.99%, -1.88%, and -2.02%. At 36 weeks, the percentage of body weight loss ranges from 3.02% to 16.94%, with the exact percentages depending on the dosage. In clinical trials, ratastride significantly improved glycemic control and significantly reduced body weight; it also had a safety profile similar to those of GLP-1 RA, GIP, and ratastride [54].

GLP-1 RA That Is Non-peptide and Administered Orally

To treat type 2 diabetes mellitus, doctors use orforglipron, a small chemical that belongs to the class of non-peptide GLP-1 RA. The pharmacokinetic profile and oral bioavailability of orforglipron were shown to be favorable in preclinical and early clinical research, ranging from 20% to 40%. Depending on the dosage, its half-life ranges from 29 to 49 hours. People who suffer from both obesity and type 2 diabetes may soon have a new treatment option in the form of orforglipron [43]. Orforglipron resulted in a -2.10% reduction in HbA1c levels (-1.67% adjusted for placebo) compared to dulaglutide's -1.10% reduction, according to a 26-week, phase 2, double-blind, randomized, multicenter study. At week 26, the average body weight had dropped by 10.1 kg, which is more than the 2.2 kg loss seen with the placebo and the 3.9 kg loss seen with dulaglutide. When compared to other GLP-1 RA at the same developmental period, the adverse event profile is very similar [55].

Basal Insulin Analog Administered Once Weekly

The major goal of controlling type 2 diabetes is glucose control, while new antidiabetic medications help with weight loss and organ preservation. When conventional antidiabetic drugs fail to control blood glucose levels in people with type 2 diabetes mellitus, insulin plays a pivotal role. Compared to prior insulin formulations, modern basal insulin formulations are very effective and are associated with a reduced risk of hypoglycemia. However, nonadherence to the daily dosage is a common problem associated with less-than-ideal glycemic control. With a low risk of hypoglycemia, weekly basal insulin injections should improve patients' quality of life, treatment adherence, and clinical inertia. Insulin icodec is a reversible and highly effective insulin analog that binds to albumin strongly because of a C20 fatty acid side chain. Insulin receptor-mediated clearance is reduced, and affinity for the insulin receptor is reduced as a consequence of this change. Over the course of the seven days of therapy, a steady-state pharmacodynamic model shows that the hypoglycemic effects are evenly distributed [56,57]. Comparing once-daily insulin glargine U100 with once-weekly insulin icodec in a phase 3a trial including 984 people with type 2 diabetes was the goal of the research. Participants were assigned to either therapy in a 1:1 ratio in this randomized, open-label, treat-to-target experiment. Between icodec and glargine U100, the average reduction in glycated hemoglobin level is 1.55% after 52 weeks, whereas the difference is 1.35%. When comparing icodec to glargine U100, the percentage of time spent in the 70-180 mg/dL range is much greater (71.9% vs. 66.9%). The rate of adverse events is similar in both groups, and no extra safety issues were observed [57].

## Conclusions

The development of new pharmacological drugs, such as inhibitors of SGLT2 and GLP-1 receptor, has resulted in significant advancements in the treatment of type 2 diabetes. In recent decades, there has been a significant increase in the number of large-scale randomized clinical studies that assess the cardiovascular safety of different drugs. Research findings have demonstrated that blocking the SGLT2 enzyme and GLP-1 receptor has positive effects on cardiovascular well-being. Several positive outcomes can be observed, such as a decreased likelihood of cardiovascular disease and overall mortality. There is a decrease in hospitalizations related to heart failure with the use of SGLT2 inhibitors and a reduced risk of stroke with GLP-1 RA. Additionally, there is a decrease in the advancement of chronic renal disease. Based on previous studies, it has been recommended by both the American Diabetes Association and the European Diabetes Association that patients with type 2 diabetes who are at risk for cardiovascular complications should consider taking metformin along with SGLT2 inhibitors and GLP-1 RA. This comprehensive treatment focuses on addressing vascular events. Both branches of medicine have their own unique contributions, despite their notable distinctions. The study reveals that GLP-1 RA has been found to reduce the likelihood of cardiovascular events, with a specific focus on stroke. On the other hand, SGLT2 inhibitors have shown promising results in alleviating heart failure. Recent recommendations from the American College of Cardiology (ACC), American College of Clinical Pharmacy (ACCP), and European Society of Cardiology (ESC) suggest that all patients suffering from heart failure should be administered medications that inhibit SGLT2. This recommendation is applicable to both individuals with and without type 2 diabetes. The literature review provides us with the necessary information to draw conclusions about the impact of SGLT2 inhibitors and GLP-1 RA on renal and cardiac outcomes in both type 2 diabetes patients and non-diabetics. We also take into account the additional advantages of GLP-1 RA and SGLT2 inhibitors for individuals with heart conditions.
